# Assessment of Rab geranylgeranyltransferase subunit beta in amyotrophic lateral sclerosis

**DOI:** 10.3389/fneur.2024.1447461

**Published:** 2024-08-19

**Authors:** Jing Yang, Mei Tian, Lei Zhang, Cheng Xin, Jia Huo, Qi Liu, Hui Dong, Rui Li, Yaling Liu

**Affiliations:** ^1^Department of Neurology, The Second Hospital of Hebei Medical University, Shijiazhuang, Hebei, China; ^2^The Key Laboratory of Neurology, Hebei Medical University, Ministry of Education, Shijiazhuang, Hebei, China; ^3^Neurological Laboratory of Hebei Province, Shijiazhuang, Hebei, China; ^4^Department of Emergency, The Second Hospital of Hebei Medical University, Shijiazhuang, Hebei, China

**Keywords:** RABGGTB, mononuclear cells-macrophages, ALS, PD, ACVD

## Abstract

**Introduction:**

Geranylgeranyltransferase Subunit Beta (RABGGTB) was expressed at higher levels in patients with Amyotrophic lateral sclerosis (ALS) compared with healthy controls. This study aims to observe the expression of RABGGTB in different cells from patients with ALS and different diseases.

**Methods:**

In this case–control study, we collected peripheral blood from patients with ALS and healthy controls, and compared the expression of RABGGTB in natural killer cells (NK), T cells and B cells between patients with ALS and healthy controls by flow cytometry. And compared the expression of RABGGTB in monocytes and monocyte-derived macrophages from patients with ALS, Parkinson’s disease (PD), acute cerebrovascular disease (ACVD), and healthy controls by flow cytometry and immunofluorescence. Then flow cytometry was used to detect the expression of RABGGTB in monocytes from SOD1G93A mice and WT mice.

**Results:**

The expression of RABGGTB was not significantly changed in NK cells, cytotoxic T cells (CTL), helper T cells (Th), regulatory T cells (Treg), and B cells from patients with ALS compared to healthy controls. And the expression of RABGGTB in monocytes and monocyte-derived macrophages was higher in the ALS group than in the PD, ACVD and control group. The expression of RABGGTB was significantly higher in monocytes of SOD1G93A mice compared to WT mice.

**Conclusion:**

These findings suggest that RABGGTB expression was increased in monocytes and monocyte-derived macrophages from patients with ALS, not in NK, CTL, Th, Treg, and B cells. Future studies are needed to find the clinical implication of RABGGTB in ALS.

## 1 Introduction

ALS is a chronic, progressive and fatal neurodegenerative disease in which the motor neurons in the anterior horn cells of the spinal cord, the brainstem, and the cerebral cortex undergo degenerative changes. About 10% of ALS cases are inherited, although the majority of cases are sporadic (1). The clinical and pathological features of sporadic and familial ALS are similar. About 20% of familial cases are associated with mutations in the Cu/Zn superoxide dismutase 1 (SOD1) gene in an autosomal dominant pattern (2). Currently, there is no known cure and few effective treatments for ALS because the exact mechanism of ALS remains elusive and the multifactorial nature of ALS includes genetic susceptibility (3), environmental exposures (4), and clinical heterogeneity (5).

Inflammation is associated with ALS. Microglia and astrocytes are key regulators of inflammatory responses (6–8), and peripheral inflammation plays an important role in ALS (9–11). Accumulating evidence suggests that inflammation including the CNS and the peripheral immune system, contributes to the progression of ALS in both humans and in mouse models (12–15). First, microglia and astroglia activation have been reported as prominent features in the spinal cord and motor cortex of ALS patients and transgenic rodent models, and microglia and astroglia have been used as therapeutic targets for ALS (16–20). Furthermore, the central and peripheral nervous systems of ALS patients and animal models have been reported to be infiltrated by macrophages, natural killer cells and T lymphocytes, and modulation these cells have been reported to improve motor function or prolong survival in ALS (10, 11, 21–24).

In recent years, several studies have focused on the role of RABGGTB in diseases. In our previous studies, we found that the expression of RabGGTase is lower in the spinal cord motoneurons in SOD1G93A mice compared with WT mice, and autophagy defects could be ameliorated by modulating RABGGTB in neurons (25), Rab geranylgeranyltransferase subunit alpha (RABGGTA) and Rab geranylgeranyltransferase subunit beta (RABGGTB) composed RabGGTase (26) that mediated the prenylation modification of Rab7, which was inhibited in the ALS model (27). In our previous studies, we have found that the expression of RABGGTB was higher in peripheral mononuclear-macrophages of ALS patients compared with healthy controls, and the expression of RABGGTB was significantly correlated with disease progression in ALS patients (28). The RABGGTB was significantly downregulated in the peripheral blood from patients with multiple sclerosis compared with healthy subjects (29), whereas high RABGGTB expression has been reported in tumor-associated disease (30).

In the present study, we first reported that the expression of RABGGTB was not significantly changed in natural killer cells (NK), cytotoxic T cells (CTL), helper T cells (Th), regulatory T cells (Treg), and B cells from patients with ALS compared to healthy controls. And the expression of RABGGTB in monocytes and monocyte-derived macrophages was higher in the ALS group than in the PD, ACVD, and control group.

## 2 Materials and methods

### 2.1 Subjects

The study was approved by the Ethics Committee of the Second Hospital of Hebei Medical University (2022-R196). Between January 2021 and August 2023, we collected medical histories and physical examination records from patients diagnosed with ALS, PD, and ACVD at the Department of Neurology. Patients included met the appropriate diagnostic criteria for ALS (31), PD (32), and ACVD (33). The patients with ALS included in the study are sporadic and depending on the site of onset, are divided into those with bulbar-onset ALS and those with limb-onset ALS. Meanwhile, the information of the corresponding healthy controls was collected from the physical examination department, including gender, age, past history, family history, and so on. All the participants with ALS, PD, and ACVD were excluded from acute or chronic inflammatory diseases, for instance, acute pneumonia, and rheumatoid arthritis. And all the participants signed informed consent forms.

### 2.2 Serum sample

Venous blood was collected from the cubital region of patients with ALS, PD, ACVD, and healthy controls in the morning after an overnight fast.

### 2.3 Flow cytometry

Erythrocyte lysis was performed by mixing 2 mL of erythrocyte lysis buffer Erythrocyte Lysis Buffer (Lysing Buffer 10X Concentrate, BD, 555899) with 1 mL of whole blood and incubating for 10 min at RT. The leukocytes were centrifuged at 500 × g for 5 min at 4°C and washed twice with PBS. The leukocytes were resuspended in 2 mL PBS and stained with antibodies specific for cell surface antigens (CD3-PE-cy7, 25-0037-42; CD4-Percp-cy5.5, 45-0049-42; CD8-eFluor450, 48-0088-42; CD56-PE, 12-0567-42; CD25-APC, 17–0257-42; CD127-PE, 12-1,278-42; CD19-APC, 17-0199-42; CD14-APC, 17-0149-42; CD16-Percp-cy5.5,46–0168-42; PE anti-mouse Ly6C, 1:100, BD, 128007; and CD45 BB515, 1:100, BD, 564590) for 30 min on ice in the dark. After washing with PBS, stained cells were fixed and permeabilized by adding 500 μL of fixation/permeabilization solution (Cytofix/CytopermTM Plus Fixation/Permeabilization Kit, BD, 554714) and incubated for 20 min at RT in the dark. The thoroughly resuspended fixed/permeabilized cells were incubated with anti-RABGGTB (1,500, GeneTeX, GTX105874) at 4°C for 30 min in the dark. The cells were then incubated again with FITC-conjugated goat anti-rabbit 1gG (H + L) (1,100, PROTEINTECH, SA00003-2) at 4°C for 30 min in the dark. After the cells were washed twice with 1 × BD Perm/WashTM buffer and resuspended in staining buffer, flow cytometry analysis was performed.

### 2.4 Isolation of PBMCs from blood samples

Human peripheral blood lymphocyte separation liquid (LTS1077) was added in a high-efficiency centrifuge tube (Tianjin Haoyang Biological Products Science & Technology Co., Ltd., Tianjin, China; 601,002), and centrifuged at 200× g for 2 min at room temperature. The peripheral blood samples were then added and centrifuged for an additional 30 min at 800× g. The intermediate mononuclear cell layer was carefully aspirated into a new centrifuge tube and centrifuged at 300× g for 13 min. The precipitate is mononuclear cells after aspiration of the supernatant.

### 2.5 *In vitro* culture of macrophages

The peripheral blood mononuclear cells were maintained in RPMI-1640 medium (Gibco, C11875500BT) supplemented with 10% FBS and 1% penicillin–streptomycin (P/S). Macrophage colony stimulating factor (M-CSF, PeproTech, 300-25-10) was added to the culture medium for 7 days to induce differentiation of monocytes into macrophages. The culture medium was changed every 3 days. On day 7, the cell culture medium was collected and fixed with 4% paraformaldehyde in PBS (34).

### 2.6 Immunofluorescence and confocal microscopy analysis

Cells were washed three times with PBS, and fixed with containing 0.3% Triton-X-100 for 15 min and blocked with 10% sheep serum for 1 h at room temperature. Then primary antibodies against CD68 (1,500, Abcam, ab31630), F4/80 (1,200, Abcam, ab6640), and RABGGTB (1,500, GeneTeX, GTX105874) were used overnight at 4°C. After washed three times, cells were incubated with the appropriate secondary antibody: Donkey anti-rat IgG (H + L) highly cross-adsorbed secondary antibody, Alexa Fluor™ 488 (1,1,000, Invitrogen, A21208), Alexa Fluor 594-conjugated goat anti-mouse secondary antibody (1,1,000, Thermo Fisher, #A-11032), and Alexa Fluor 647-labeled goat anti-rabbit secondary antibody (1,1,000, Invitrogen, A21245) for 1 h at room temperature. Cells were observed using a fluorescence confocal microscope (LSM900, ZEISS, Germany). Microscope parameters were set at the beginning of each imaging session and remained constant throughout the imaging session.

### 2.7 Animals and treatments

SOD1G93A mice (B6SJL-Tg [SOD1G93A]1Gur/J) were originally obtained from Jackson Laboratory (Bar Harbor, ME, United States). DNA was extracted from the tails of hemizygous mice and genotyped by PCR. Mice were housed under constant temperature (22–24°C), constant humidity (40–60%) and 12 h light/dark cycle and fed with sterilised water and aseptic granular food. Mice were deeply anesthetized by pentobarbital sodium and blood was collected from the heart of mice. All studies were conducted in accordance with the Guidelines for the Management of Laboratory Animals formulated by the Ministry of Science and Technology of China, and the Animal Ethics Committee of the Second Hospital of Hebei Medical University also approved the experimental procedures (Approval no. 2023-R248). Throughout the experiment, we did our best to reduce the suffering of all animals.

### 2.8 Statistical analysis

Statistical analyses and graphing were performed using GraphPad Prism 9 (GraphPad software). All data were presented as mean ± SD, and performed with skewness and kurtosis tests for normal distribution check. For data with normal distribution, unpaired *t*-test was used for differences analysis between patients with ALS and healthy controls, and paired *t-*test were used between SOD1G93A mice and WT mice. For abnormal distributed data, Mann–Whitney *U-*test was used for the differences analysis. *p* ≤ 0.05 was considered as statistically significant.

## 3 Results

### 3.1 Expression of RABGGTB in different cells from patients with ALS and healthy control

In our previous studies, we have found that the expression of RABGGTB was higher in peripheral mononuclear-macrophages of ALS patients compared with healthy controls (28), so we want to compare the RABGGTB levels in other cells from patients with ALS and healthy controls. So, we collected 43 patients diagnosed with ALS at the Second Hospital of Hebei Medical University between January 2021 and August 2023. The healthy control group consisted of 20 male and seven female with a mean age of 57 ± 6 years, and ALS group included a total of 32 male and 11 female with a mean age of 58 ± 7 years. The distributions of age and gender did not differ significantly (Table 1).

**Table 1:** Demographic parameters of healthy controls and ALS patients.

Variables	Healthy controls	ALS patients	*P*
Participants (number)	27	43	
Age (years)	57 ± 6	58 ± 7	>0.05
Sex (Female/male)	7/20	11/32	>0.05
Site of onset			
Bulbar	NA	9	
Limb	NA	34	

HC, healthy control; ALS, amyotrophic lateral sclerosis.

**Figure 1:**
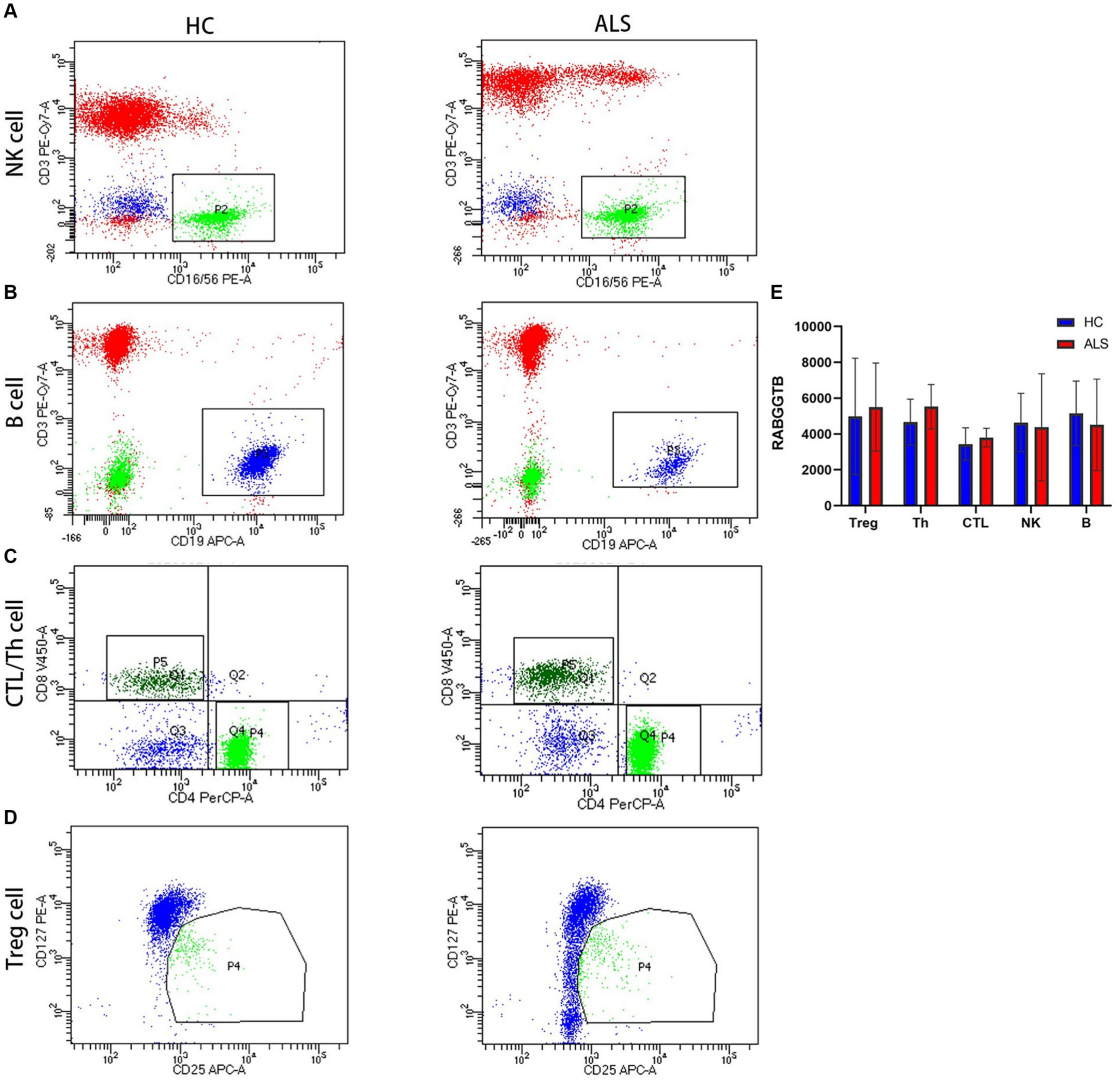
Evaluation of the RABGGTB level in different cells from patients with ALS and healthy controls. **(A)** RABGGTB levels in NK cells from patients with ALS and healthy controls by BD flow cytometry. **(B)** RABGGTB levels in B cells from patients with ALS and healthy controls by BD flow cytometry. **(C)** RABGGTB levels in CTL and Th cells from patients with ALS and healthy controls by BD flow cytometry. **(D)** RABGGTB levels in Treg cells from patients with ALS and healthy controls by BD flow cytometry. **(E)** Quantitative analysis of the RABGGTB level in different cells. The statistical significance was determined using an unpaired *t*-test. ^*^p ≤ 0.05, ^**^p ≤ 0.01, ^***^p ≤ 0.001, ^****^p ≤ 0.0001. HC, healthy control; ALS, amyotrophic lateral sclerosis; RABGGTB, Rab geranylgeranyltransferase subunit beta.

T Cells were divided into three subpopulations based on the level of CD3 CD4 CD8 CD25 and CD127 surface expression: Treg cell (CD4 + CD25 + CD127-), Th cell (CD3 + CD4 + CD8-), and CTL cell (CD3 + CD4-CD8+). Flow cytometry was used to detect RABGGTB levels in natural killer cells (NK), cytotoxic T cells (CTL), helper T cells (Th), regulatory T cells (Treg), and B cells from patients with ALS compared with healthy controls (Figures 1A–D). Comparing the expression levels of RABGGTB between the groups, we discovered that RABGGTB was not significantly changed in peripheral natural killer cells (NK), cytotoxic T cells (CTL), helper T cells (Th), regulatory T cells (Treg), and B cells of patients with ALS (Figure 1E).

### 3.2 Expression of RABGGTB in the monocytes from patients and healthy control

Combining the above results, we hypothesized whether the increased expression of RABGGTB in monocytes from patients with ALS is specific. Therefore, we included two neurological diseases, including patients with PD and ACVD, as controls. The data of 90 patients diagnosed with ALS, 35 patients diagnosed with PD, 20 patients diagnosed with ACVD at the Second Hospital of Hebei Medical University between January 2021 and August 2023, were collected. The healthy control group consisted of 34 male and 16 female with a mean age of 56 ± 8 years, and ALS group included a total of 62 male and 28 female with a mean age of 59 ± 9 years, and PD group consisted of 23 male and 12 female with a mean age of 57 ± 8 years, and ACVD group included a total of 13 male and seven female with a mean age of 55 ± 8 years. The distributions of age and gender did not differ significantly (Table 2).

**Table 2:** Summary of donor information.

Variables	Healthy controls	ALS patients	PD patients	ACVD patients	*p*-value
Participants (number)	50	90	35	20	
Age (years)	56 ± 8	59 ± 9	57 ± 8	55 ± 8	*p* > 0.05
Sex (Female/male)	16/34	28/62	12/23	7/13	*P* > 0.05

HC, healthy control; ALS, amyotrophic lateral sclerosis; PD, Parkinson’s disease; ACVD, acute cerebrovascular disease.

**Figure 2:**
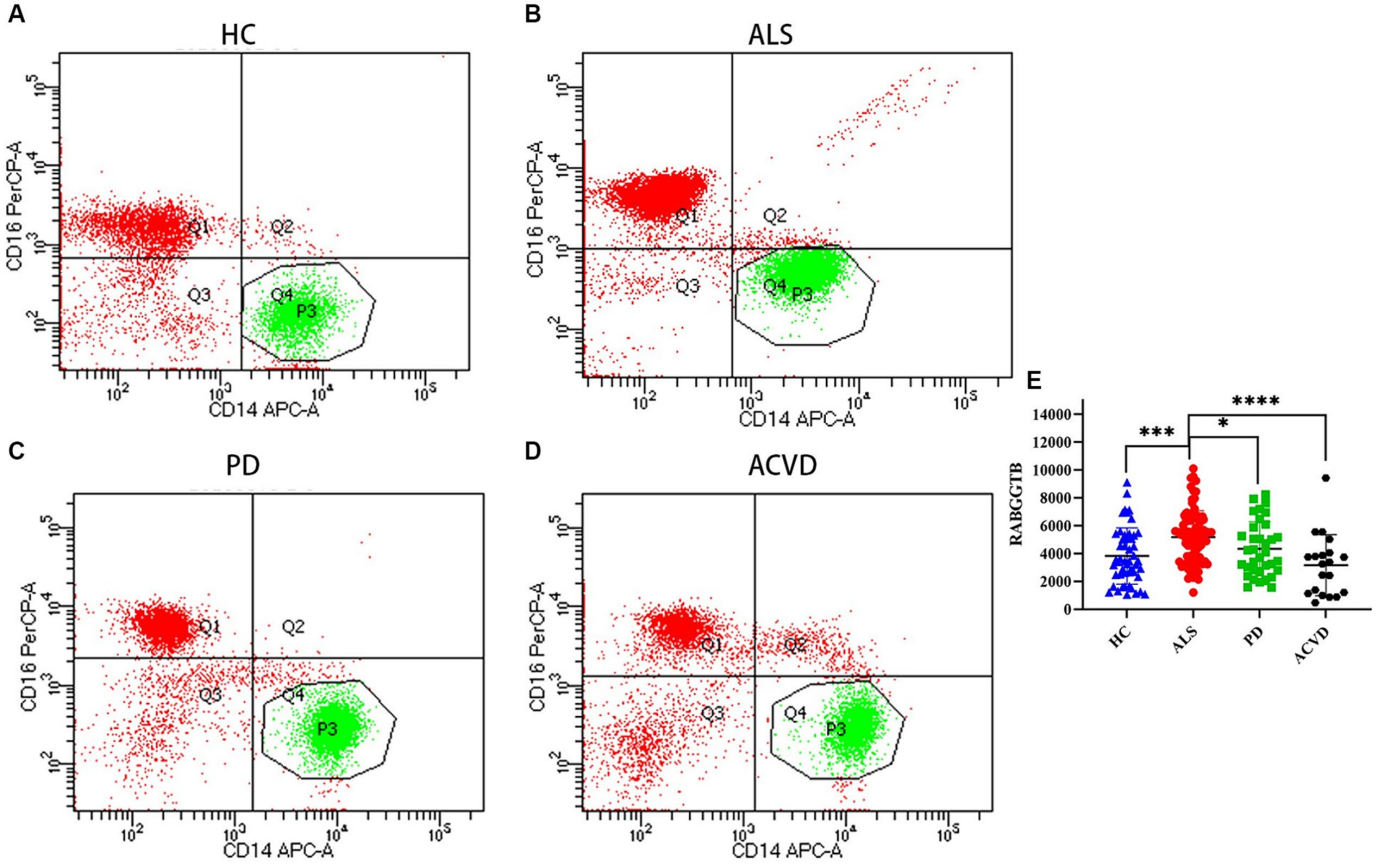
Evaluation of the RABGGTB level in classical monocytes from patients with ALS, PD, ACVD, and healthy controls. **(A)** RABGGTB levels in monocytes from healthy controls by BD flow cytometry. **(B)** RABGGTB levels in monocytes from ALS patients by BD flow cytometry. **(C)** RABGGTB levels in monocytes from PD patients by BD flow cytometry. **(D)** RABGGTB levels in monocytes from ACVD patients by BD flow cytometry. **(E)** Quantitative analysis of the RABGGTB level in monocytes. The statistical significance was determined using an unpaired *t*-test. ^*^p ≤ 0.05, ^**^p ≤ 0.01, ^***^p ≤ 0.001, ^****^p ≤ 0.0001. HC, healthy control; ALS, amyotrophic lateral sclerosis; PD, Parkinson’s disease; ACVD, acute cerebrovascular disease; RABGGTB, Rab geranylgeranyltransferase subunit beta.

BD flow cytometry was used to detect the RABGGTB levels in the monocytes from ALS patients, PD patients, ACVD patients, and healthy controls (Figures 2A–D). We compared the expression of RABGGTB in monocytes between the four groups, and we discovered that RABGGTB was significant highly upregulated in monocytes of patients with ALS (Figure 2E).

### 3.3 Expression of RABGGTB in the monocyte-derived macrophages from patients and healthy control

**Figure 3:**
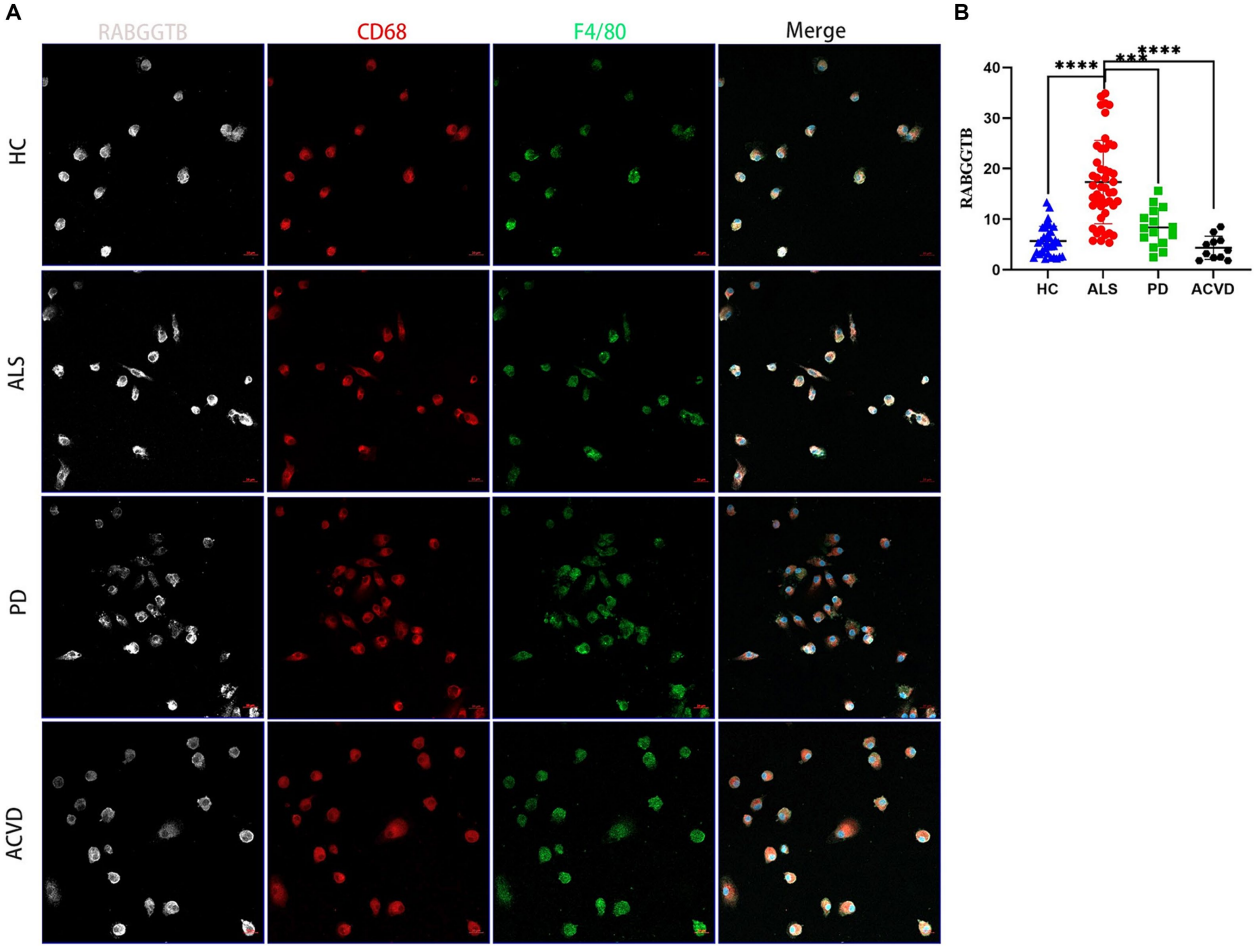
Evaluation of the RABGGTB level in monocytes-derived macrophages from patients with ALS, PD, ACV and healthy controls. **(A)** Immunofluorescence labeling for RABGGTB (gray/white), CD68 (red), and F4/80 (green) in monocyte-derived macrophages from patients and healthy controls. DAPI was used to stain nuclei (blue). Scale bars = 20 μm. **(B)** Quantitative analysis of RABGGTB levels in monocyte-derived macrophages from patients and healthy controls. The statistical significance was determined using an unpaired *t*-test. ^*^p ≤ 0.05, ^**^p ≤ 0.01, ^***^p ≤ 0.001, ^****^p ≤ 0.0001. HC, healthy control; ALS, amyotrophic lateral sclerosis; PD, Parkinson’s disease; ACVD, acute cerebrovascular disease; RABGGTB, Rab geranylgeranyltransferase subunit beta.

In order to further validate this result, the monocytes extracted from peripheral blood were differentiated into macrophages after stimulated by M-CSF. The cells presented typical macrophages morphology, with macrophage markers CD68 and F4/80 expressed on the cell surface (Figure 3A). We discovered that the expression of RABGGTB in monocyte-derived macrophages was significantly higher in ALS group than that in the other three groups (Figure 3B).

### 3.4 The expression of RABGGTB increased in the monocytes of SOD1G93A

**Figure 4:**
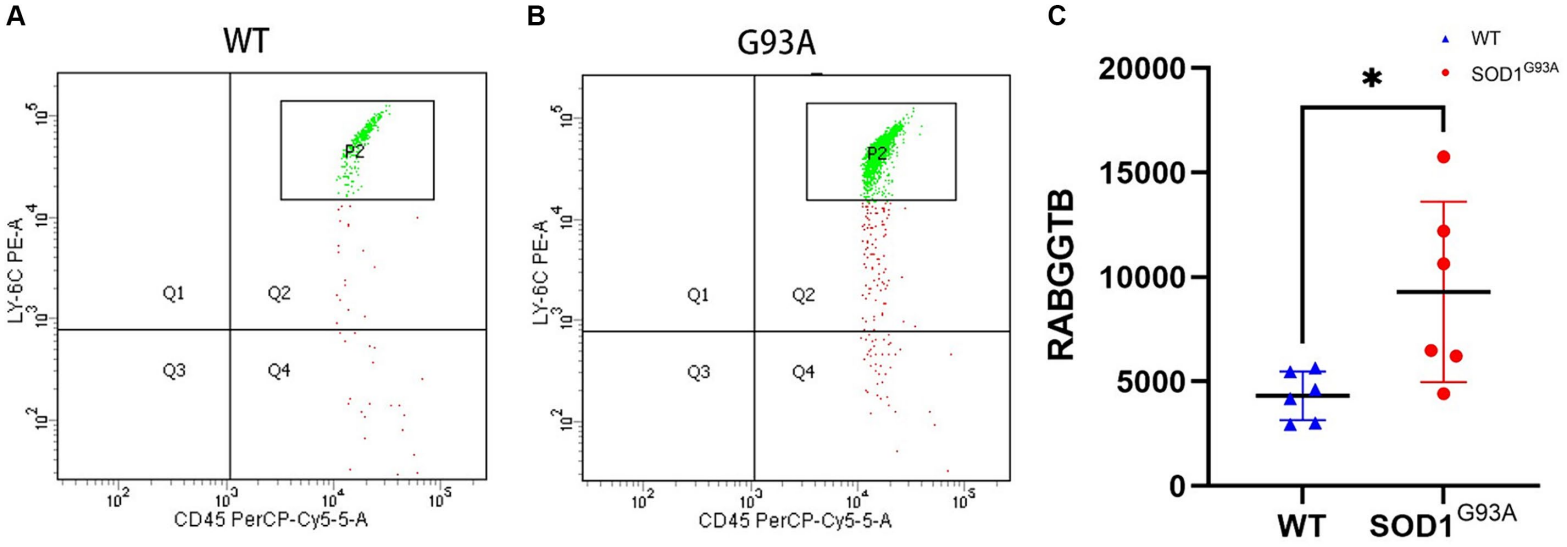
Evaluation of the RABGGTB level in monocytes of SOD1G93A and WT mice. **(A)**. RABGGTB levels in LY6C-positive and CD45-positive monocytes of WT mice by BD flow cytometry. **(B)**. RABGGTB levels in LY6C-positive and CD45-positive monocytes of SOD1G93A mice by BD flow cytometry. **(C)**. Quantitative analysis of the RABGGTB level in monocytes. The statistical significance was determined using an unpaired *t*-test. ^*^p ≤ 0.05, ^**^p ≤ 0.01, ^***^p ≤ 0.001, ^****^p ≤ 0.0001. RABGGTB, Rab geranylgeranyltransferase subunit beta.

The above findings suggest that the expression of RABGGTB in monocytes and macrophages was significantly higher in the ALS group than in the other groups, but the data collected come from sporadic ALS, and these data are not representative of familial ALS (fALS), such as those with mutations in the Cu/Zn-superoxide dismutase (SOD1) gene. In order to detect the expression of RABGGTB in monocytes with mutations in the SOD1 gene, we used flow cytometry to detect the expression of RABGGTB in monocytes of SOD1G93A mice, comparing the expression levels of RABGGTB in monocytes of WT mice (Figures 4A,B), we discovered that the expression of RABGGTB was significantly upregulated in monocytes of SOD1G93A mice (Figure 4C).

## 4 Discussion

Inflammation plays a key role in the development and progression of ALS, and chronic inflammation can contribute to progressive neuron loss (35–37). The study of inflammation and ALS has been going on for decades, but few anti-inflammatory treatments have reached the clinic (37). Previous research has shown that different inflammatory cells play different roles in ALS, and our previous studies found that the expression of RABGGTB was higher in peripheral mononuclear-macrophages from ALS patients (28), in this study, we discovered that the expression levels of RABGGTB was not significantly changed in peripheral NK, CTL, Th, Treg, and B cells between patients with ALS and healthy controls. And the expression of RABGGTB in peripheral monocyte and monocyte-derived macrophages was significantly higher in the ALS group than in the PD group, ACVD group and the control group. These findings suggested that it might be specific that the high expression of RABGGTB in peripheral monocyte and monocyte-derived macrophages from patients with ALS.

The expression of RABGGTB was significantly high in peripheral monocyte and monocyte-derived macrophages from patients with ALS, but the data collected come from sporadic ALS, and these data are not representative of familial ALS (fALS), such as those with mutations in the Cu/Zn-superoxide dismutase (SOD1) gene, so we detect the expression of RABGGTB in monocytes of SOD1G93A mice and found that the expression of RABGGTB was significantly upregulated in monocytes of SOD1G93A mice. These findings show that the expression of RABGGTB is increased in peripheral blood mononuclear macrophages from ALS patients and SOD1G93A mice.

Taken together, these results suggest that perhaps the high expression of RABGGTB in ALS may have a specificity, but further experiments are necessary for verification. Of course, these results raise the question why the RABGGTB expression is increased in monocytes from patients with ALS. Our speculation is that the main reason may be that the inflammatory states of ALS are different from the other diseases. A second reason is the different nature and pathogenesis of the diseases. However, the mechanisms involved in this are not clear, and we will be investigating these mechanisms in future studies. Inflammation plays an important role in ALS and there has been a lot of research on anti-inflammatory drugs in ALS, such as celecoxib and minocycline (38–40). However, few anti-inflammatory drugs in ALS have reached the clinic. Our results may suggest that some indicators or functions of inflammatory cells in ALS are different from the other diseases, and this may be one of the reasons why anti-inflammatory drugs have been not reached the clinic in ALS.

Although our results are interesting, there are some limitations in the study. The main limitation is that it may be a coincidence that we only observed high expression of RABGGTB in ALS. Furthermore, there may be a selection bias among the other diseases as controls. The Second limitation is that the number of patients included was limited. ALS is an adult-onset, rare, severe neurodegenerative disease with a median survival of 3–5 years. Cases are therefore difficult to recruit, resulting in relatively small sample sizes. The third limitation is that patients with ALS have not been genetically tested for the presence of SOD1 mutations. Therefore, we used the SOD1G93A mice as a proxy for SOD1 mutation and observed that the expression of RABGGTB is increased in SOD1 mutant mice, but these data are not fully representative of humans. The four limitation is that we did not carry out a mechanistic study of the effect. However, this research is ongoing and we will be investigating the mechanisms in a future study, and future studies are needed to find the clinical implication of RABGGTB in ALS.

In conclusion, our data demonstrate that it might be specific that the high expression of RABGGTB in peripheral monocyte and monocyte-derived macrophages from patients with ALS, further emphasizing the contribution of monocyte s to physiological processes. These findings provide additional evidence for peripheral immune cells to participate in ALS, and provide a new insight for peripheral immunotherapy in ALS, and then we will further verify in the follow-up study.

## Data Availability

The original contributions presented in the study are included in the article/supplementary material, further inquiries can be directed to the corresponding authors.
